# Assessing relational coordination and its impact on perceived mental health of students, teachers and staff in a clinical skills program during the COVID-19 pandemic

**DOI:** 10.1186/s12909-022-03828-3

**Published:** 2022-11-10

**Authors:** Ali S. Alfazari, Hebatallah A. Naim Ali, Awad Alessa, Mohi Eldin Magzoub

**Affiliations:** 1 Medical Simulation Center, College of Medicine & Health Sciences, United Arab Emirates University, Al Ain, United Arab Emirates; 2 The Heller School for Social Policy and Management, Brandeis University, Waltham Massachusetts, USA; 3 Department of Medical Education, College of Medicine & Health Sciences, United Arab Emirates University, Al Ain, United Arab Emirates

**Keywords:** Clinical skills, Medical education, Relational coordination, COVID-19, Mental health, Work engagement, Job satisfaction, Burnout

## Abstract

**Background:**

The global spread of the COVID-19 virus caused unprecedented interruptions in medical education. This paper evaluates Relational Coordination (RC): communicating and relating for task integration; between the distinct stakeholders responsible for scheduling,delivering and receiving clinical teaching in the wake of the pandemic.

**Methodology:**

Using a cross-sectional design, the level of Relational Coordination was assessed between twelve groups within a Clinical Skills Program at a Medical School in the United Arab Emirates. It also measures three relevant mental health factors: namely, Job satisfaction, Work Engagement, and Burnout.

**Results:**

Overall, RC scores were moderate (3.65 out of 5.00). Controlling for participants' position, RC was found to positively and significantly increase both job satisfaction (β = 1.10, *p* < 0.001) and work engagement (β = 0.78, *p* < 0.01)., Additionally, RC was significantly associated with lower burnout (β = -0.56, *p* = 0.05). Fifty percent of participants experienced high job satisfaction, with a mean score of 5.0 out of 7.0, while 73% reported being enthusiastic about their job, with a mean score of 6.0 out of 7.0. About a third of participants (27%) reported feeling burnout.

**Conclusions:**

During times of disruption and crisis, medical education can benefit from higher levels of relational coordination. Our study shows the significant impact of relational coordination on mental health measures like job satisfaction and work engagement. To achieve the full potential and benefits of excellent levels of relational coordination in this program, we recommend six interventions focusing on improving communication, work processes, regular meetings, education innovations, capacity building, and the establishment of coaching and counseling programs for students and faculty.

**Supplementary Information:**

The online version contains supplementary material available at 10.1186/s12909-022-03828-3.

## Background

The fundamental mission of medical schools is to prepare students to address the health needs of society. As a prerequisite for clinical clerkships, preclinical medical students learn physical diagnostic and communication skills at inhouse simulation and skills labs in medical schools. Multiple interdependent teams and departments from within the medical school and affiliated teaching facilities work together to deliver the preclinical skills program. The supervision of learners, staff development, alignment of stakeholders, operational flexibility, and educational evaluation are known challenges in clinical skills training programs [1]. Better across departmental integration and coordination of training activities is necessary for the progressive and professional development of medical students as future healthcare providers [2].

The COVID-19 pandemic has markedly influenced medical education across the globe. Teaching clinical skills during the pandemic was a global challenge [3]. New methods of curriculum delivery tools such as distance learning and simulation technologies were utilized with mixed perceptions from students and faculty. In a recent survey, 62.2% of medical students in South Korea were happy with online teaching, and 84.3% preferred to continue online education after the COVID-19 pandemic [4]. On the other hand, 43.3% of preclinical students in the US felt unprepared for clinical clerkships due to lack of clinical skills teaching, laboratory, and hands-on training [5].

Relational coordination theory proposes that relationships of shared goals, shared knowledge, and mutual respect help to support frequent, timely, accurate, problem-solving communication and vice versa [6]. High-quality relational coordination is associated with resilience and problem-solving skills, leading to better project outcomes [7]. Moreover, relational coordination has increased satisfaction with university e-learning programs amongst students and instructors [8]. In fact, high-level relational coordination, satisfaction with communication and work environment were inversely associated with turnover and intention to stay [9, 10]. In addition to job satisfaction, relational coordination was associated with additional staff outcomes, namely less burnout and greater work engagement [11].

In higher education organizations, better educational outcomes are achieved through high-quality interprofessional communication and sharing knowledge and goals in an environment of mutual respect [8]. A recent survey of relational coordination in higher education institutions in Pakistan showed weak relational coordination in cross-teams work integration and collaboration [12].

People with greater work-life balance performed better when their psychological well-being was reinforced by their satisfaction with their co-workers [13]. Furthermore, perceptions of peer justice increase staff's job satisfaction by strengthening the effect of high-performance work systems on relational coordination among workers [14].

In this paper, we assess the level of relational coordination and its impact on perceived mental health factors in a Clinical Skills Program in the United Arab Emirates during the peak of the COVID-19 pandemic in 2020. We hypothesized that the relational coordination experienced by stakeholders in the Clinical Skills Program can predict mental health factors.

## Methods

### Ethical approval

This study was approved by the United Arab Emirates University Social Sciences Research Ethics Committee: Reference No. ERS_2021_7313. All participants provided informed consent for the study.

## Setting

The study was conducted at the College of Medicine and Health Sciences, United Arab Emirates University (UAEU). The Clinical Skills Program is a 12-credit hour mandatory 2-year program. Third- and fourth-year medical students complete the program before joining clinical clerkships at affiliated clinical teaching sites [15]. Third year students have to progress through the program to get promoted to fourth year. Fourth year medical students should be ready for clerkships the following year. The course comprises ten modules of 6 to8 weeks duration and is conducted at the Medical Simulation Center. In each module, students learn a specific set of clinical skills ranging from medical interviewing and communication skills to physical diagnostics and bedside clinical procedures. Two course directors oversee the entire course assisted by two coordinators for each module. The directors, coordinators and clinical tutors come from diverse clinical departments within the college and affiliated hospitals and clinics. Multiple interdependent teams and departments within the college and off-campus worked together to deliver the course.

In the wake of the COVID-19 pandemic, the initial lockdowns and strict measures imposed by the UAE health authorities caused unprecedented disruptions at the medical school. Lectures were delivered online and virtually. However, clinical skills teaching required face-to-face interactions and hands-on sessions training. Therefore, a hybrid model of education was adopted by the college to resume the Clinical Skills Program. More than 12 teams and workgroups had to work together to continue to deliver the course including the course directors, coordinators, faculty, staff, local COVID-19 committee, simulated patients, and medical students. Meticulous arrangements were made to organize the students groups and pair them with clinical tutors while making sure schedules did not clash and that all staff and students were compliant with the COVID-19 precautions.

As the number of COVID-19 cases climbed across the country, clinical faculty struggled with the increased load of clinical duties at hospitals and the complicated teaching schedules at the medical school. Staff and faculty relied heavily on email communications and occasional virtual meetings to keep the program running. Unessential staff were asked to work from home to reduce the infection risk. The Medical Simulation Center imposed strict infection control procedures such as mandatory negative nasal swab tests, social distancing, and reduced teaching space capacities. Students were divided into smaller groups which required more teaching slots that needed to be filled by faculty and simulated patients. Faculty and staff were exhausted as schedules were adjusted almost weekly. Faculty struggled with the unfamiliar virtual teaching tools and frequently required more IT support. Uncertainty and anxiety caused mistrust in the hybrid teaching model. The morale of many students, staff and faculty was down, and a sense of helplessness ensued.

**Fig. 1:**
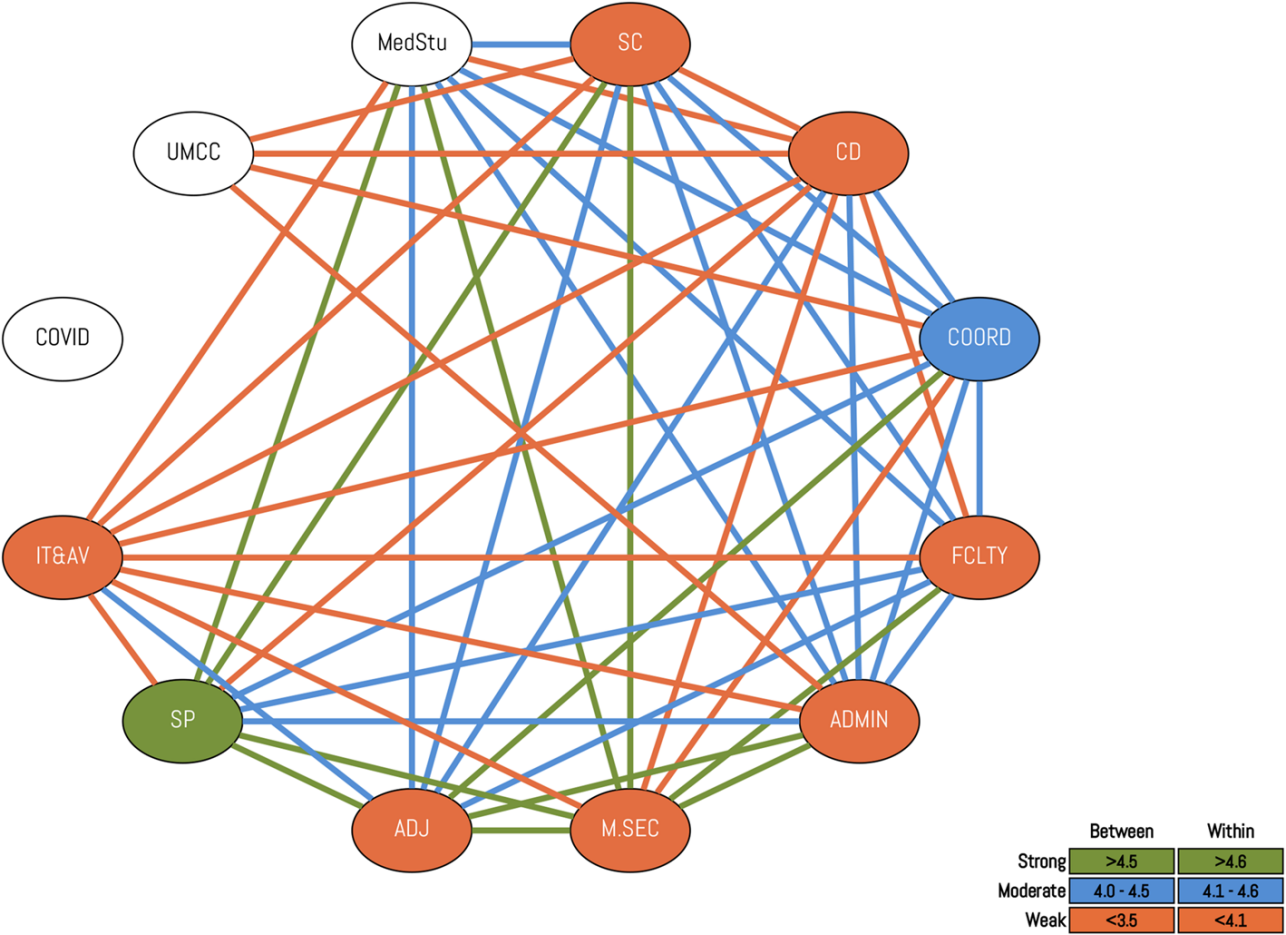
Relational map of the Clinicals Skills Program, 2020 (The colors within the circles signify the strength of reported relational coordination among participants of the same role (within ties), while the colors of lines connecting the different circles signify the strength of relational coordination between participants across different roles (between ties). The colors within the circles signify the strength of reported relational coordination among participants of the same role (within ties), while the colors of lines connecting the different circles signify the strength of relational coordination between participants across different roles (between ties). SC: Simulation Staff; CD: Course Directors; COORD: Module Coordinators; FCLTY: Clinical Faculty; ADMIN: Administrative Staff; M.SEC: Medical Secretaries; ADJ: Adjunct Clinical Faculty; SP: Simulated Patients; IT&AV: IT and Audiovisual Support Team; COVID: COVID-19 Steering Committee; UMCC: Undergraduate Medical Curriculum Committee; MedStu: Medical Students)

To help understand the coordination levels within the Clinical Skills Program, the relevant roles and workgroups engaged in administering the program were mapped. Figure 1 shows the complex, interdependent and cross-cutting coordination needed to run the program in 2020.

### Participants and survey

All eligible participants from the twelve selected roles; including students, staff, course managers, and faculty, were invited to participate in a 7-item validated Relational Coordination Survey (RCS). The Relational Coordination Survey (RCS) is a short 7-item instrument that asks participants to rate each dimension of the relational coordination between them and other roles in the workplace on a 5-point scale. The RCS is a fully validated instrument whose unbounded property makes it applicable across multiple levels- cross-professional, cross-unit, cross-organization, and between providers and clients [16, 17].The analytics generated from the RCS reveal the strength of relational coordination networks among all the interdependent interprofessional teams, as experienced by participants. Since English was the language of instruction in the clinical skills course, the RCS survey was implemented in its original English version. The reliability coefficient- Cronbach's alpha- for the RCS in our sample was 0.91. Table 1 shows the mean and standard deviation of the total RC score and its seven dimensions and their correlation with other studied outcomes.

**Table 1:** Summary and correlation of Relational Coordination and other studied outcomes

**Variables**	**N**	**Mean (SD)**	**1**	**2**	**3**	**4**	**5**	**6**	**7**	**8**	**9**	**10**
**1. Total RC**	79	3.64 (0.94)	^**−**^	^**−**^	^**−**^	^**−**^	^**−**^	^**−**^	^**−**^	^**−**^	^**−**^	^**−**^
2. Frequent Comm	77	3.93 (1.00)	0.80^***^	^**−**^	^**−**^	^**−**^	^**−**^	^**−**^	^**−**^	^**−**^	^**−**^	^**−**^
3. Timely Comm	71	3.46 (1.07)	0.89^***^	0.73^***^	^**−**^	^**−**^	^**−**^	^**−**^	^**−**^	^**−**^	^**−**^	^**−**^
4. Accurate Comm	68	3.66 (1.15)	0.90^***^	0.69^***^	0.76^***^	^**−**^	^**−**^	^**−**^	^**−**^	^**−**^	^**−**^	^**−**^
5. Problem-Solving Comm	59	4.04 (0.80)	0.85^***^	0.52^***^	0.65^***^	0.67^***^	^**−**^	^**−**^	^**−**^	^**−**^	^**−**^	^**−**^
6. Shared Goals	61	3.53 (1.07)	0.81^***^	0.48^***^	0.63^***^	0.66^***^	0.70^***^	^**−**^	^**−**^	^**−**^	^**−**^	^**−**^
7. Shared Knowledge	57	3.61 (1.08)	0.83^***^	0.44^***^	0.68^***^	0.64^***^	0.69^***^	0.64^***^	^**−**^	^**−**^	^**−**^	^**−**^
8. Mutual Respect	58	3.98 (1.01)	0.80^***^	0.39^**^	0.57^***^	0.64^***^	0.72^***^	0.63^***^	0.69^***^	^**−**^	^**−**^	^**−**^
**9. Job Satisfaction**	62	4.98 (1.73)	0.54^***^	0.37^**^	0.47^***^	0.47^***^	0.51^***^	0.42^***^	0.55^***^	0.53^***^	^**−**^	^**−**^
**10. Work Engagement**	64	5.69 (1.75)	0.36^*^	0.24 ^NS^	0.37^**^	0.31^*^	0.25 ^NS^	0.28^*^	0.43^***^	0.36^**^	0.74^***^	^**−**^
**11. Burnout**	64	3.77 (2.03)	-0.19^NS^	-0.20 ^NS^	-0.17 ^NS^	-0.10 ^NS^	-0.12 ^NS^	-0.34^**^	-0.28^*^	-0.01 ^NS^	-0.14 ^NS^	-0.29^*^

*NS* No statistical significance

^*^p < 0.05

^**^p < 0.01

^***^p < 0.001

In addition to assessing relational coordination, participants were also asked to assess their job satisfaction, work engagement, and burnout levels. These three aspects of participant well-being were assessed using a 7-point Likert-type scale, ranging from 1 (never experienced) to 7 (experienced every day). Job satisfaction and work engagement are positive scales, where higher scores indicate greater well-being. In contrast, burnout is a negative scale, where higher scores indicate poor mental health [Appendix A].

### Analytic methods

Survey data were analyzed using STATA17. Proportional weights were created to adjust the regression models for lower response rates for specific roles, ensuring that the results reflect the sampled population. Given that the 12 roles contributed differently to the scheduling and operation of the skills course, a new variable "Participants Position" was generated to classify the roles into three categories: management and administration, teaching staff, and medical students. Table 2 shows the survey response rate and position for each of the twelve roles.

**Table 2:** The response rate among the different stakeholders participating in the survey

**Stakeholders Role**	**Position**	**Invited**	**Completed**	**Response rate**
1. Academic Secretaries	**Management & administration**	7	1	14%
2. Adjunct Faculty	**Teaching Faculty**	28	2	7%
3. Clinical Faculty	**Teaching Faculty**	21	14	67%
4. Course Administrators	**Management & administration**	2	1	50%
5. Course Directors	**Management & administration**	2	2	100%
6. COVID Committee	**Management & administration**	2	0	0%
7. IT & Audiovisual Support	**Management & administration**	4	1	25%
8. Medical Students	**Medical Students**	170	29	17%
9. Module Coordinators	**Management & administration**	14	5	36%
10. Simulated Patients	**Teaching Faculty**	6	4	67%
11. Simulation Center	**Management & administration**	5	5	100%
12. UG Medical Curriculum Committee	**Management & administration**	**3**	**1**	**33%**
**Total**		**264**	**65**	**25%**

## Results

Eighty-two participants responded out of 264 invited. Sixty-five participants completed the survey. The overall response rate was 25%. Response rates varied across the different roles; adjunct faculty (7%), academic secretaries (14%), course students (17%), IT & audiovisual support (25%), medical curriculum committee (33%), module coordinators (36%), course administrators (50%), clinical faculty (67%), simulated patients (67%). The lowest participation rate was from the COVID committee, with none of the invited members completed the survey. On the other hand, all invited participants from the simulation center and course directors' workgroups have completed the survey with a response rate of 100%.

### Relational coordination

Across all roles involved in the clinical skills course, relational coordination (RC) was moderate, with a total RC index of 3.65 out of 5.00. Mutual respect was the strongest dimension reported by participants with a total score of 4.01, followed by problem-solving communication and frequent communication (total scores of 3.95 and 3.92, respectively). The strongest RC between roles was reported with the simulation center, which received a score of 4.13, where the strongest dimensions were frequent, timely, accurate, and problem-solving communication (4.37, 4.06, 4.15, 4.22, respectively). The weakest RC between roles was reported with the undergraduate medical curriculum committee (3.27 out of 5.0) and medical students (3.35 out of 5.0). Academic secretaries reported the strongest RC ties with other roles, with a total RC of 4.61 out of 5.00, followed by simulated patients with a total RC of 4.54 out of 5.00. The undergraduate medical curriculum committee, course directors, and the simulation center experienced weaker RC ties with other roles (overall reported RC indices were3.07, 3.13, 3.31, respectively).

### Burnout, satisfaction and work engagement

**Fig. 2:**
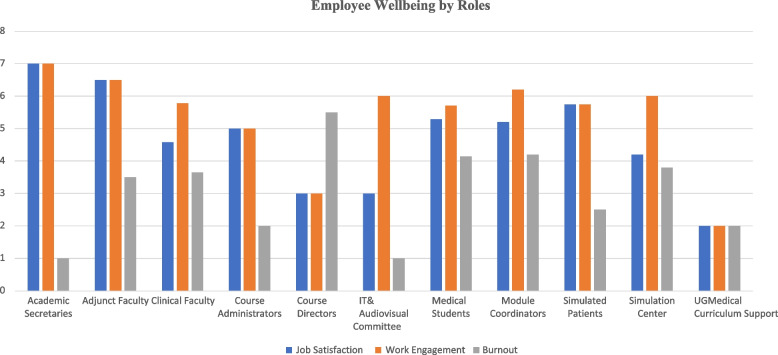
Well-being scores reported by each participating role

Fifty percent of participants reported experiencing high job satisfaction every day or a few times a week, with a mean score of 5.0 out of 7.0, while 73%of participants reported being enthusiastic about their job every day or a few times a week, with a mean score of 6.0 out of 7.0. About a third of participants (27%) reported feeling burnout "*almost every day*" or *“a few times a week,*" with a total mean of 3.8 out of 7.0. Comparing across roles, academic secretaries reported the highest job satisfaction and work engagement and the lowest burnout level, while course directors and module coordinators reported the highest level of burnout. Also, medical students reported high levels of burnout despite reporting high work engagement and job satisfaction. Figure 2 shows the well-being scores reported by each participating role.

### Impact of relational coordination on mental health factors

Multivariate regression models were conducted to predict the impact of relational coordination on each of the studied outcomes: burnout, job satisfaction, and work engagement. Controlling for participants' position, RC total index was found to positively and significantly increase job satisfaction (β=1.1, *p* <0.001). Problem-solving communication had the highest impact (β=1.15, *p *<0.001). Table 3 summarizes the regression models for RC Total Index and its seven dimensions on participants' job satisfaction.

**Table 3:** Regression models for predicting employee job satisfaction^a^

*Variables*	**Job Satisfaction**							
	Model1	Model2	Model3	Model4	Model5	Model6	Model7	Model8
**Total RC**	_1.1_***	-	-	-	-	-	-	-
Frequent Comm	-	0.69^**^	-	-	-	-	-	-
Timely Comm	-	-	0.77^***^	-	-	-	-	-
Accurate Comm	-	-	-	0.67^**^	-	-	-	-
Problem-Solving Comm	-	-	-	-	1.15^***^	-	-	-
Shared Goals	-	-	-	-	-	0.72^***^	-	-
Shared Knowledge	-	-	-	-	-	-	0.83^***^	-
Mutual Respect	-	-	-	-	-	-	-	0.88^**^
Participants’ position								
Management & Administration	Reference	Reference	Reference	Reference	Reference	Reference	Reference	Reference
Teaching Staff	0.28 ^NS^	0.79^*^	0.45 ^NS^	0.38 ^NS^	0.14 ^NS^	0.43 ^NS^	0.46 ^NS^	0.22 ^NS^
Medical Students	0.26 ^NS^	0.60 ^NS^	0.46 ^NS^	0.24 ^NS^	0.19 ^NS^	0.50 ^NS^	0.05 ^NS^	0.08 ^NS^
**Constant**	0.85^NS^	1.94^*^	2.14^**^	2.51^***^	0. 39^NS^	2.26^**^	2.10^**^	1.58^NS^
**Adjusted *R***^**2**^	0.33	0.17	0.22	0.22	0.31	0.23	0.30	0.28
**F-TEST(*P*-VALUE)**	11.87^***^	6.16^***^	5.84^**^	11.90^***^	7.70^***^	5.29^**^	7.84^***^	4.47^**^

*NS* No statistical significance

^*^p < 0.05

^**^p < 0.01

^***^p < 0.001

^a^The regression models used proportional weights to adjust for lower response rate among certain groups

Aside from frequency and accuracy of communication, all RC dimensions were also positively and statistically associated with higher work engagement. Work engagement was also positively and significantly associated with relational coordination total index (β = 0.78, *p* < 0.01). Table 4 summarizes the regression models for Relational Coordination Total Index and its seven dimensions on participants' work engagement.

**Table 4:** Regression models for predicting employee work engagement^a^

*Variables*	**Work Engagement**							
	Model1^a^	Model2^a^	Model3^a^	Model4^a^	Model5^a^	Model6^a^	Model7^a^	Model8^a^
**Total RC**	0.78^**^	-	-	-	-	-	-	-
Frequent Comm	-	0.41^NS^	-	-	-	-	-	-
Timely Comm	-	-	0.60^**^	-	-	-	-	-
Accurate Comm	-	-	-	0.42 ^NS^	-	-	-	-
Problem-Solving Comm	-	-	-	-	0.72^*^	-	-	-
Shared Goals	-	-	-	-	-	0.48^*^	-	-
Shared Knowledge	-	-	-	-	-	-	0.76^**^	-
Mutual Respect	-	-	-	-	-	-	-	0.71^***^
Participants’ position								
Management & Administration	Reference	Reference	Reference	Reference	Reference	Reference	Reference	Reference
Teaching Staff	0.02 ^NS^	0.46 ^NS^	0.17 ^NS^	0.16 ^NS^	-0.27 ^NS^	0.34 ^NS^	0.43 ^NS^	0.53 ^NS^
Medical Students	-0.19 ^NS^	0.05 ^NS^	-0.05 ^NS^	-0.18 ^NS^	-0.01 ^NS^	0.21 ^NS^	-0.13 ^NS^	-0.12 ^NS^
**Constant**	2.92^**^	4.02^***^	3.68^***^	4.30^***^	2.88^NS^	3.86^***^	3.04^**^	3.03^***^
**Adjusted** *R*^**2**^	0.17	0.07	0.16	0.10	0.14	0.10	0.27	0.20
**F-TEST(*P*-VALUE)**	3.24^*^	1.36^NS^	2.84^*^	1.69 ^NS^	1.60 ^NS^	1.56 ^NS^	5.10^**^	4.59^**^

*NS* No statistical significance

^*^p < 0.05

^**^p < 0.01

^***^p < 0.001

^a^The regression models used proportional weights to adjust for lower response rate among certain groups

The regression model showed that relational coordination was also associated with lower burnout (β=-0.56, *p*=0.05). Moreover, there was a statistically significant association between shared goals and shared knowledge and lower burnout levels (β=-0.63, β=-0.77, respectively). The models showed that medical students experienced significantly higher burnout compared to participants in management and administrative roles. Table 5 summarizes the regression models assessing the impact of total relational coordination and its seven dimensions on participants' burnout levels.

**Table 5:** Regression models for predicting employee burnout^a^

*Variables*	**Burnout**							
	Model1^a^	Model2^a^	Model3^a^	Model4^a^	Model5^a^	Model6^a^	Model7^a^	Model8^a^
**Total RC**	-0.56^*^	-	-	-	-	-	-	-
Frequent Comm	-	-0.42^NS^	-	-	-	-	-	-
Timely Comm	-	-	-0.41^NS^	-	-	-	-	-
Accurate Comm	-	-	-	-0.24^NS^	-	-	-	-
Problem-Solving Comm	-	-	-	-	-0.43^NS^	-	-	-
Shared Goals	-	-	-	-	-	-0.63^**^	-	-
Shared Knowledge	-	-	-	-	-	-	-0.77^**^	-
Mutual Respect	-	-	-	-	-	-	-	-0.11^NS^
Participants’ position								
Management & Administration	Reference	Reference	Reference	Reference	Reference	Reference	Reference	Reference
Teaching Staff	0.66 ^NS^	0.50 ^NS^	0.70 ^NS^	0.67 ^NS^	-0.06 ^NS^	-0.37 ^NS^	0.10 ^NS^	-0.29 ^NS^
Medical Students	1.30^**^	1.12^*^	1.20^**^	1.27^**^	0.87 ^NS^	0.59 ^NS^	1.09^*^	1.02 ^NS^
**Constant**	5.00^***^	4.68^***^	4.36^***^	3.76^***^	4.88^**^	5.61^***^	5.90^***^	3.54^***^
**Adjusted *R***^**2**^	0.11	0.09	0.09	0.07	0.08	0.18	0.21	0.07
**F-TEST(*P*-VALUE)**	3.84^*^	3.15^*^	3.87^*^	2.58^NS^	1.38^NS^	5.27^**^	4.52^**^	1.90^NS^

*NS* No statistical significance

^*^p < 0.05

^**^p < 0.01

^***^p < 0.001

^a^The regression models used proportional weights to adjust for lower response rate among certain groups

## Discussion

Overall, participants in this Clinical Skills Program experienced moderate relational coordination with “mutual respect” dimension being the strongest. Well-defined roles and responsibilities within our medical school may explain the elevated levels of mutual respect amongst participants in various roles despite all the hardships experienced by the academic and administrative staff as well as the students in this program. On the other hand, relationships dimensions of “shared goals” and “shared knowledge” were weak. In comparison, three dimensions of communication (frequent, accurate, and problem-solving) were moderately strong, except “timely communication” which was the weakest dimension across all participants. This was evident as there were gaps in communication especially related to teaching cancelations and rescheduling due to the uncertainly and the evolving health regulations at the beginning of the pandemic. Simulation center staff reported moderate relational coordination amongst themselves, scoring the weakest on shared goals. They all have distinct but complementary tasks that range from classroom setup to training and supervising simulated patients. This could be explained by the initial pandemic related confusion and struggle to try to keep the skills program running while mitigating the infection risks and restrictions. In pre-COVID-19 era, the clinical skills program schedules would have been approved at the beginning of the academic year with limited changes during the course.

There was a wide variation in the relational coordination experienced by participants. Our analyses showed how these variations impacted participants ‘well-being in significant ways. In particular, the Course Directors and Module Coordinators reported the highest burnout levels due to the constant stress, the physical and mental demands of running a 2-year program during a global pandemic There was extreme sense of anxiety within the program leadership team and the medical school. Interruptions of the program could lead to delay in progression of medical students through their education which would have negative implications on supply of much needed healthcare providers workforce. . In addition to their responsibilities within the clinical skills program, the course directors and module coordinators were often tasked to assist or cross cover for additional administrative, teaching, and clinical duties.

Evidence from prior studies suggest that improving relational coordination across the board can lead to favorable employee occupational outcomes such as reduced burnout and emotional exhaustion [11, 18]. In our study, relational coordination was found to positively and significantly increased job satisfaction and work engagement and was marginally associated with lower burnout. These results are in keeping with prior well-established research in which high-quality relationships and communications promote emotional and physical well-being [19]. There was a strong will and courage within the college community at large to keep the medical education programs running to eventually meet the country’s need for more healthcare providers.

Though learning outcomes were not measured in this study, it is understood that lower levels of relational coordination would significantly impact learning outcomes, based on the relational coordination theory [6] and from previous research [15, 20–22].

Medical students reported the highest levels of burnout as they had to deal with uncertainty, unpredictable class cancelations, difficulty rescheduling missed sessions, and an overall dislike of the online teaching model in medical education. These findings mirror similar recent studies that reported high-stress levels by medical students in Saudi Arabia, with 22.3% of students stating severe stress after introduction of online learning [23].

### Practice implications

**Fig. 3:**
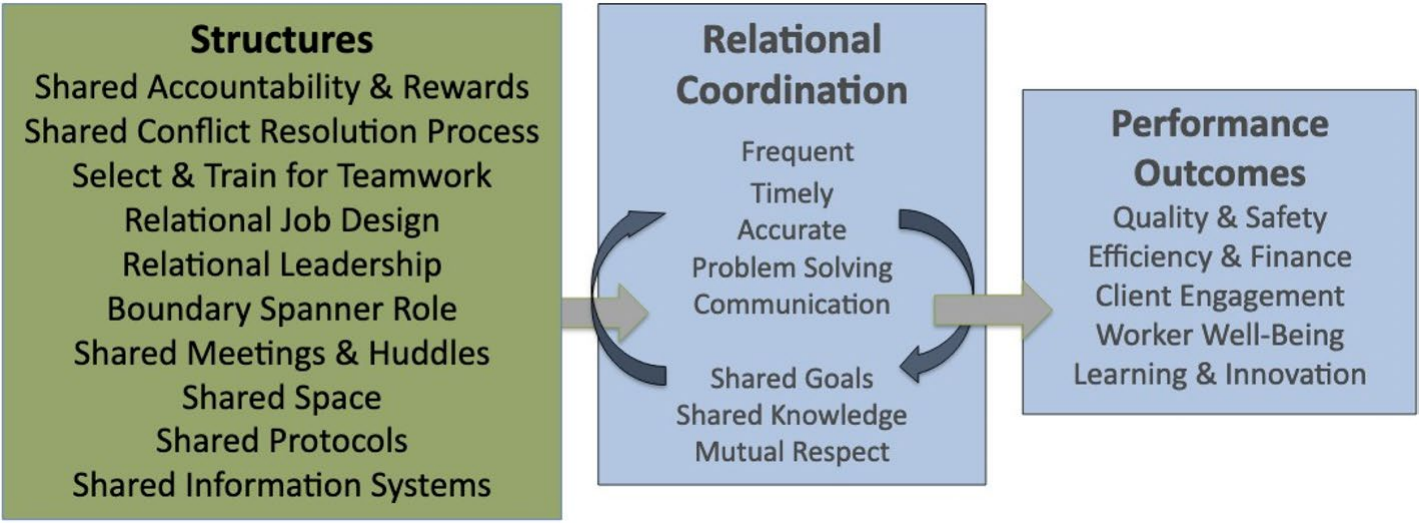
Structure-process-outcomes model of relational coordination

Relational coordination theory has been tested in 74 industry contexts such as airlines, banking, pharmaceuticals, software, financial services, the criminal justice system, multiple levels of education including higher education, and many areas of healthcare, and in 37 countries [6]. To our knowledge, this is the first study that assessed relational coordination and its impact on stakeholders’ mental health in a clinical skills program in the COVID-19 pandemic. The results of this study clearly show a moderate level of relational coordination between all groups. Although many participants reported positive well-being, 27% of participants still reported high levels of burnout. According to the Relational Model of Organizational Change [24], relational coordination can be strengthened by adopting organizational structures such as shared accountability for outcomes, shared reward systems, shared meetings, shared work protocols, and shared information systems, as well as hiring and training staff to engage in interdependent teamwork. Figure 3: shows structure-process-outcomes model of relational coordination.

Based on our findings, we propose the following interventions to improve the work environment atthe clinical skills program at times of crisis. First, create a bidirectional closed-communication platform for the clinical skills course, accessible by all participating teams, including students to foster shared information systems. Effective communication at times of crisis is crucial for institutions to mitigate risks associated with unpredictability and extraordinary situations and develop resilience [25]. Second, hold regular structured student-faculty meetings with teams across multiple units within the college, including senior college leadership. These meetings should focus on streamlining work processes, addressing issues with clear role definitions, tracking outcomes, and ensuring accountability amongst all workgroups. Third, provide targeted training focused on interprofessional teamwork to improve relational coordination, as seen in previous studies [11]. Given the necessity for interprofessional teamwork in clinical practice, these training opportunities should be available to all team members and medical students.

Fourth, adopt creative and innovative solutions such as the use of modern learning management systems as well as increasing the utilization of new teaching methods such as medical simulation programs [26] and introduction of early clinical exposure of medical students to the clinical setting [27].

Fifth, incorporate medical residents and students' peer teaching in the course. One of the major issues encountered during the pandemic was the shortage of available clinical tutors due to unprecedented clinical workload. Clinical clerks appreciate teaching by residents, which brings about greater satisfaction in clinical rotations [28]. Moreover, utilizing a pool of dedicated medical residents to teach basic clinical skills to preclinical medical students will be an excellent option to improve the supply of future clinical tutors for the course. The college could also use a select group of senior medical students to teach and supervise preclinical students learning clinical skills. These practices have been used in many medical schools as students tend to learn better from their seniors, especially in small group structured sessions in clinical skills [29].

Finally, create a college-wide coaching and counseling program for education managers, directors, coordinators, and faculty and staff is essential to focus on resilience and crisis management skills and provide enough support for faculty members. Ongoing coaching and counseling programs will support the course directors, coordinators, and faculty members by polishing their managing skills at times of crisis [30]. Table 6 summarizes the proposed interventions.

**Table 6:** Suggested interventions to improve the work environment at the clinical skills program at times of crisis

**Intervention**	**Description**	**Desired outcomes**
1. Communication platform	Creating college-wide effective bi-directional communication platform for administrative, teaching and students	Effective communication at times of crisis is crucial for institutions to mitigate risks associated with unpredictability and extraordinary situations
2. Regular structured meetings	Holding regular student-faculty meetings with teams across multiple units within the college, including senior college leadership	Streamlining work processes, addressing issues with clear role definitions, tracking outcomes, and ensuring accountability amongst all workgroups
3. Interprofessional teamwork training	Workshops focusing on interprofessional teamwork	Improve relational coordination among teams and workgroups
4. Innovative education management	Adopting new creative and innovative approaches in medical education such as high-fidelity medical simulation programs and introduction of early clinical exposure of medical students	Reduce dependency on constant pool of teaching faculty and allow more self-directed learning of clinical skills
5. Peer teaching programs	Encourage peer teaching and supervision in clinical skills programs	Reduce the need for full time faculty and motivation of students as they learn from their role model peers
6. Coaching and counseling programs	Establishing coaching and counseling programs for education managers, directors, coordinators, and faculty and staff	Support the course education managers, and faculty members by polishing their administrative and stress management skills at times of crisis and beyond

### Limitations

Our findings should be interpreted with caution due to several limitations worth mentioning. First, the study is cross-sectional, which limits evidence of causality. Secondly, the study has a small sample from a single academic institution. Replication of this study in multiple academic institutions would be of high interest. Thirdly, the study was conducted in May 2020, which coincided with the holy month of Ramadan in the United Arab Emirates. This might explain low response rates, especially for specific roles as the COVID- 19 committee members and Undergraduate Medical Curriculum Committee. Fourthly, demographic data were not available alongside the relational coordination and mental health factors. It would be important to explore some variability in participants’ experiences with the hybrid teaching model for different age and gender groups. Finally, the program’s impact and success were not measured in this study which should be addressed in future studies.

## Conclusion

Using a cross-sectional design, this study assessed relational coordination and its impact on three mental health measures (i.e. job satisfaction, work engagement, and burnout) between 12 integral roles and stakeholders in a Clinical Skills Program in a Medical School in the United Arab Emirates,. Our medical college has managed to continue curriculum delivery of the Clinical Skills Program despite challenges imposed by the COVID-19 pandemic. The participants in this Clinical Skills Program experienced moderate relational coordination with many participants reported positive occupational well-being. Yet, 27% of participants still reported high levels of burnout. Relational coordination was found to positively and significantly increase job satisfaction, work engagement, and marginally lower burnout. Practice-based interventions are proposed to foster higher relational coordination with the aim of improving students and staff wellbeing within the Clinical Skills Program at times of crisis and disruption. 

## Supplementary Information

**Additional file 1.**

## Data Availability

The datasets generated during and/or analyzed during the current study are available from the corresponding author on reasonable request.
