# Supplementary material for: Assessing relational coordination and its impact on perceived mental health of students, teachers and staff in a clinical skills program during the COVID-19 pandemic
Source: BMC Med Educ. 2022 Nov 10;22:773. doi: 10.1186/s12909-022-03828-3 (PMC9647241; doi:10.1186/s12909-022-03828-3)
Supplement: Supplementary file 1 — Additional file 1. [file 12909_2022_3828_MOESM1_ESM.docx]

**Appendix A**

| **Constructs** | **Measures** | **Response Anchors** | **Sources** | **Validation Status** |
| --- | --- | --- | --- | --- |
| Frequent Communication | How frequently do people in each of these groups communicate with you about the scheduling of Clinical Skills Teaching? | Not nearly enough (1) to just the right amount () | Gittell, 2009 | Validated as part of the  RC Index |
| Timely Communication | Do they communicate with you in a timely way about the scheduling of Clinical Skills Teaching? | Never (1) to always (5) | Gittell, 2009 | Validated as part of the  RC Index |
| Accurate communication | Do they communicate with you accurately about the scheduling of Clinical Skills Teaching? | Never (1) to always (5) | Gittell, 2009 | Validated as part of the  RC Index |
| Problem Solving Communication | When there is a problem with the scheduling of Clinical Skills Teaching, do people in each of these groups blame others or work with you to solve the problem? | Always blame (1) to always solve (5) | Gittell, 2009 | Validated as part of the  RC Index |
| Shared Goals | Do people in each of these groups share your goals for the scheduling of Clinical Skills Teaching? | Not at all (1) to completely (5) | Gittell, 2009 | Validated as part of the  RC Index |
| Shared Knowledge | Do people in each of these groups know about the work you do with the scheduling of Clinical Skills Teaching? | Nothing (1) to everything (5) | Gittell, 2009 | Validated as part of the  RC Index |
| Mutual Respect | Do people in each of these groups respect the work you do with the scheduling of Clinical Skills Teaching? | Not at all (1) to completely (5) | Gittell, 2009 | Validated as part of the  RC Index |
| **Relational Coord Index** | *An equally weighted index of the previous five measures* | 1 to 5 | Gittell, 2009 | Validated as part of the  RC Index |
| **Job satisfaction** | How often do you agree with the following statement:  My level of job satisfaction is high. | Never (1) to every day (7) | Nagy, Mark S., 2002 | Validated as a single measure by Scarpello and Campbell, (1983) |
| **Work Engagement** | How often do you agree with the following statement:  I am enthusiastic about my job. | Never (1) to every day (7) | Maslach, CH et al, 2001 | Not previously validated as a stand-alone measure |
| **Burnout** | How often do you agree with the following statement:  I feel burned out from my job. | Never (1) to every day (7) | Maslach, CH et al, 2001 | Not previously validated as a stand-alone measure |

Gittell, Jody Hoffer. *High Performance Healthcare: Using the Power of Relationships to Achieve Quality, Efficiency and Resilience*. McGraw-Hill, 2009. Print.

Nagy, Mark S. “Using a Single-Item Approach to Measure Facet Job Satisfaction.” *Journal of occupational and organizational psychology* 75.1 (2002): 77–86. Web.

Maslach, CH et al. “Job Burnout.” *Annual review of psychology* 52.1 (2001): 397–422. Web.
